# Acute effects of whole body vibration on foot sole sensitivity and plantar pressures during gait initiation

**DOI:** 10.1186/1757-1146-5-S1-O24

**Published:** 2012-04-10

**Authors:** Martin Alfuth, Anne Beiring, Dieter Klein, Dieter Rosenbaum

**Affiliations:** 1 Movement Analysis Laboratory, Institute of Experimental Musculoskeletal Medicine (IEMM), University Hospital Muenster (UKM), Domagkstr. 3, 48149 Muenster, Germany

## Background

Sensory receptors in the skin of the foot sole show a site-specific sensitivity to local pressures and vibrations [1] and provide feedback during foot loading activities. Impaired plantar feedback has been shown to affect plantar pressures and kinematics during gait [2-5]. The present study investigated the acute effects of whole body vibration on plantar sensitivity and foot loading during gait.

## Materials and methods

Fifteen healthy subjects (28.4 ± 4.4 years) were tested before and after 3 minutes of whole body vibrations at a frequency of 30 Hz (bilateral stance on a Galileo^®^ Med M Plus vibration trainer with slightly bent knees). Semmes-Weinstein monofilaments were used to test plantar sensitivity to light touch at the hallux and the heel. Plantar pressures during gait initiation were recorded using an EMED-ST4 platform.

## Results

**Figure 1:**
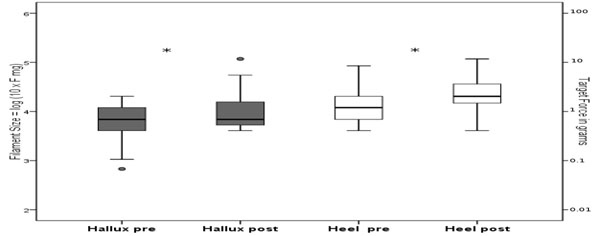
Boxplots with plantar sensitivity thresholds of the hallux and the heel before and after whole body vibration (*p < 0.025).

Plantar sensitivity thresholds were significantly increased after whole body vibration (p < 0.025), i.e. a decreased plantar sensitivity was observed under the heel (5.8%) and the hallux (7.1%; Fig. 1). No significant changes were found in plantar pressure parameters during gait initiation.

## Conclusions

In conclusion, high-intensity whole-body vibration affects plantar sensitivity by slightly increasing the sensory perception thresholds. However, this decrease in plantar feedback does not seem to be functionally relevant with respect to foot loading during gait initiation.
